# New perspectives to improve critical care benchmarking

**DOI:** 10.1186/s13613-018-0363-0

**Published:** 2018-02-02

**Authors:** Jorge I. F. Salluh, Jean Daniel Chiche, Carlos Eduardo Reis, Marcio Soares

**Affiliations:** 1 Department of Critical Care and Graduate Program in Translational Medicine, D’Or Institute for Research and Education, Rua Diniz Cordeiro, 30 – 3° andar, Rio de Janeiro, RJ CEP 22281-100 Brazil; 2 Programa de Pós-Graduação em Clínica Médica, Universidade Federal do Rio de Janeiro, Rio de Janeiro, Brazil; 3 Institut Cochin, INSERM U1016, CNRS UMR8104, Paris, France; 4 Université Paris Descartes, Sorbonne Paris Cité, Paris, France; 5 Réanimation Médicale, Hôpital Cochin, Hôpitaux Universitaires Paris Centre, Assistance Publique–Hôpitaux de Paris, Paris, France; 6 Epimed Solutions, Rio de Janeiro, Brazil

**Keywords:** Big data, Benchmarking, Mortality, Intensive care, Quality improvement, Scoring systems

## Introduction

 In the last decades, experts and medical societies have strongly recommended the measurement of quality indicators and the assessment of intensive care unit (ICU) performance [1, 2]. This recommendation is based on the concept that measurement will generate transparency of results for multiple stakeholders and be a robust source of targets for quality improvement to be translated into improved clinical outcomes. Apart from the measurement of the results of a single ICU over time, reproducible and risk-adjusted outcomes are used to generate performance comparisons [3] in benchmarking projects for ICUs as employed in hospitals networks and national registries [4].

### What’s next for critical care benchmarking?

**Fig. 1:**
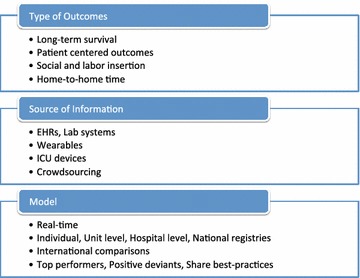
The future of critical care benchmarking

The widespread use of information technology (IT) in healthcare altogether with more sophisticated modeling statistics and newer techniques such as machine learning have potential implications for the next steps in benchmarking (Fig. 1). From the IT perspective, increased interoperability of medical devices, electronic health records (EHRs) and information systems will change the amount and speed of data acquisition and presentation to healthcare professionals, additionally it will expand access to data on processes of care and patient-centered outcomes. Moreover, the widespread use of mobile and wearable devices will allow access to information regarding quality of life, activities of daily living and functional status reported by the patients. In a recent study involving 60,000 ICU admissions, functional capacity prior to ICU admission was not only independently associated with outcomes but also improved the prognostic ability of SAPS3 [5]. Web-based centralized systems and mobile apps will play a major role in this scenario as shown in recent studies of post-hospital follow-ups [6] and survivors of critical illness. This should allow a better evaluation of the effectiveness of ICU care on long-term survival, readmissions, social and labor insertion and cognitive function. Also, as the healthcare systems evolve with the increasing role of long-term acute care facilities, hospices and institutions dedicated to rehabilitation, outcomes such as hospital length of stay may become obsolete for benchmarking. Thus, thinking of a full cycle of an episode of care, outcomes such as “home-to-home” time can be pursued and benchmarked.

Finally, as machine learning and cluster analysis are applied in critical care, they will also facilitate benchmarking. These techniques will enable clinicians to better understand the clinical profiles of critically ill populations without being necessarily categorized a priori by a diagnosis or a level of severity. In studies performed in large databases of real-world patients [7] or clinical trials [8], patients with the same diagnosis and comparable severity of illness have different profiles, response to treatments and outcomes. Therefore, an expected next step for these applications will be to test them for benchmarking purposes as they could reduce the heterogeneity of the critically ill populations enabling even more objective comparisons of outcomes.

### Actionable data: a way to decrease practice variation

The expression “Actionable data” has been used in the context of quality improvement initiatives. Let’s use the low-tidal volume (Vt) in ARDS, a practice associated with reduced mortality and shorter ICU length of stay (LOS), as an example. At patient level, daily checklists using mobile devices could show the current recommendation (Vt ≤ 6 mL/Kg IBW) and capture the patients’ current tidal volume, as well as the average data in the ICU and in the country/region thus helping to improve adherence to good standards of care. The aggregate of all the information captured by the checklist could be used at unit level for a better understanding of its overall adherence rate and comparing it with similar ICUs and ICU populations. Data available in a dashboard allows ICUs to compare the overall rates and specific outcomes (ICU LOS, duration of MV, VAP rates) and also to analyze what are the patterns of ICUs that are top ranked. These positive outliers could be considered as “positive deviants.” This methodology is used with success in other medical areas and briefly consists of understanding what are the characteristics of patients (e.g., case-mix, severity, etiology of ARF), structure (e.g., staffing patterns, types of ICU) and processes of care (e.g., sedation practices, weaning protocols, ventilator settings) of those that achieve better results. This may provide a road map for a PDCA cycle based on benchmarking of practices that are associated with better performance.

Through national ICU registries, benchmarking would support the implementation of public health policies in critical care and reduce practice variation and to increase efficiency. Currently, several countries have successfully implemented national ICU registries. Some initiatives as those in the UK (www.icnarc.org), Netherlands (www.stichting-nice.nl), Brazil (www.utisbrasileiras.com) and Australia–New Zealand (www.anzics.com.au) provide open access to epidemiologic data on critical illness, outcomes and resource utilization in these countries.

With the increasing implementation of EHRs and the availability of multiple data sources, projects are starting to collect information on processes of care. The availability of adherence to best practices, outcomes and evidence-based recommendations may decrease practice variation and improve outcomes. The NICE project has recently introduced this concept in relevant clinical areas such as pain control, red blood cell transfusion triggers or the use of antimicrobials where it is feasible to implement and perhaps reduce the evidence to practice gaps in implementation [9].

Also, increasingly, our community questions the impact of traditional clinical trials on our ability to change practice and improve patient outcome. The IT components of modern healthcare organizations and benchmarking projects may foster the development of “platform” trials, allowing to focus on diseases or syndromes, comparing multiple interventions within different domains of treatment [10]. The next leap could be the advent of a new kind of randomized, embedded, multifactorial, adaptive platform (REMAP) trials that will leverage the strengths of big data while retaining the advantages of randomization [11]. Perhaps combining elements of clinical research and continuous quality improvement programs, these trials may fuse clinical research and quality improvement.

## Conclusion

In conclusion, benchmarking is a reliable tool for quality improvement. The recent advances in IT and data science will change the way ICU benchmarking is provided, making information widely available and actionable to improve patient’s outcomes.

## Abbreviations

ICU: intensive care unit
IT: information technology
EHR: electronic health records
Vt: low-tidal volume
LOS: length of stay
VAP: ventilator-associated pneumonia
ARF: acute respiratory failure
MV: mechanical ventilation
NICE: Netherlands Intensive Care Evaluation
REMAP: randomized, embedded, multifactorial, adaptive platform trial
